# Association of Dietary Insulin Index and Dietary Insulin Load With Metabolic Health Status in Iranian Overweight and Obese Adolescents

**DOI:** 10.3389/fnut.2022.821089

**Published:** 2022-03-17

**Authors:** Zahra Hajhashemy, Saeideh Mirzaei, Ali Asadi, Masoumeh Akhlaghi, Parvane Saneei

**Affiliations:** ^1^Department of Community Nutrition, School of Nutrition and Food Science, Food Security Research Center, Isfahan University of Medical Sciences, Isfahan, Iran; ^2^Students' Research Committee, Isfahan University of Medical Sciences, Isfahan, Iran; ^3^Department of Community Nutrition, School of Nutrition and Food Sciences, Shiraz University of Medical Sciences, Shiraz, Iran; ^4^Department of Exercise Physiology, School of Physical Education and Sport Sciences, University of Tehran, Tehran, Iran

**Keywords:** obesity, metabolic health status, adolescents, dietary insulin load (DIL), dietary insulin index (DII)

## Abstract

**Background:**

Limited data are available on the association of dietary insulin load (DIL) and dietary insulin index (DII) with health status in pediatrics. We aimed to investigate the relationship of DIL and DII with metabolic health status in Iranian overweight/obese adolescents.

**Methods:**

In this cross-sectional study, using a multistage cluster random-sampling method, 203 overweight/obese adolescents (aged 12 to <18 years) were included. A validated 147-item food frequency questionnaire (FFQ) was used for a dietary intake assessment. Glycemic and lipid profile, blood pressure (BP), and anthropometric indices were measured. Participants were categorized as metabolically healthy obese (MHO) or metabolically unhealthy obese (MUO) using the two methods of the International Diabetes Federation (IDF) and a combination of IDF with Homeostasis Model Assessment Insulin Resistance (HOMA-IR).

**Results:**

According to IDF and IDF/HOMA-IR strategies, 38.9% (*n* = 79) and 33.0% (*n* = 67) of participants belonged to MUO category. After adjustments for potential confounders, subjects in the highest tertile of DIL in comparison with those in the lowest tertile had the odds ratio (OR) values of 8.44 (95% CI: 2.24–31.78) and 5.86 (95% CI: 1.39–24.58) for MUO based on IDF and IDF/HOMA-IR definitions, respectively. Moreover, after considering potential confounders, adolescents in the highest tertile of DII, compared to the lowest tertile, were, respectively, 6.93 (OR: 6.93; 95% CI: 2.59–18.57) and 5.26 (OR: 5.26; 95% CI: 1.85–14.97) times more likely to be MUO, based on IDF and IDF/HOMA-IR definitions. A significant decreasing trend was observed for OR of MUO in tertiles of DIL and DII. The stratified analysis revealed that these associations were stronger in obese participants; in overweight subjects, the association was not independent of confounders.

**Conclusion:**

This population-based study revealed that higher DIL and DII were strongly related to increased OR of MUO in Iranian adolescents, especially in obese participants. Further investigations, especially with a prospective design, are needed to affirm these findings.

## Introduction

Recent decades have witnessed a rise in the prevalence of obesity among children and adolescents worldwide. This condition has been attributed to abnormal or excessive fat accumulating in the body (1, 2). According to Global Burden of Disease (GBD) data, by the year 2025, there will be 268 million overweight children and 124 million obese adolescents globally (3). Nearly four million Iranian children and adolescents are expected to be overweight by 2025 (4). Adolescents with high body mass index (BMI) tend to become obese in their adulthood (5). Moreover, obesity in early life has been associated with cardiometabolic disorders, such as high blood pressure (BP), dyslipidemia, insulin resistance (IR), and impaired glucose metabolism, even in children (6, 7). In addition, obesity in children can increase the risk of cardiovascular disease (CVD) and all-cause mortality in adulthood (8). In turn, this condition has an economic impact on the healthcare systems (9). Metabolically healthy overweight or obese (MHO) children have a favorable cardiometabolic profile and higher insulin sensitivity, whereas metabolically unhealthy overweight or obese (MUO) subjects have a less favorable metabolic profile (10, 11).

Metabolic health statuses would be converted to each other (10). With increasing age, the percentage of individuals with MHO status drops almost linearly (12). Identifying the factors that influence metabolism in childhood is critical in preventing and treating adverse cardiometabolic diseases (13). The interaction of genetic and lifestyle factors, including diet and physical activity, may determine metabolic health status (14) and may play a role in shifting from MHO to MUO, later in life (10). Among dietary factors, carbohydrates are the main macronutrient that could stimulate postprandial secretion by increasing blood glucose (15). Nevertheless, besides blood glucose, some fatty acids and special amino acids are involved in the insulinogenic effects of foods (15–17). The food insulin index (FII) shows the quantity of postprandial insulin after consumption of each food (18). FII of each food is the ratio of insulin response after consumption of that food to insulin response of isoenergetic reference food (either glucose or white bread) in healthy subjects (18). In addition, dietary insulin load (DIL) and dietary insulin index (DII) are two indices that represent insulin response to the whole diet (19, 20).

Several previous studies have investigated the association between DII and DIL and metabolic disorders in adults. These investigations have documented positive significant associations between DII and DIL and odds ratio (OR) of metabolic syndrome (MetS) (21), obesity (22), and type 2 diabetes mellitus (T2D) (23). However, they could not find significant associations between DIL and DII and risk of CVD in adults (24). In the case of pediatric population, a cross-sectional study in children and adolescents documented that DII and DIL, especially for breakfast and dinner, were related to elevated OR of overweight (25).

To the best of our knowledge, there was no study that examined the relationship between DII and DIL with metabolic health status in pediatrics. We aimed to investigate the relationship of DIL and DII with metabolic health status in Iranian overweight/obese adolescents. To obtain enough MUO cases and investigate their relationship with DII and DIL, we selected our participants from overweight and obese adolescents.

## Materials and Methods

### Participants and Study Design

The current cross-sectional study was conducted on a representative sample of adolescents living in Iran in 2020. The sample size of this study was calculated based on previously published investigations (26, 27), which showed that approximately 60% of overweight and obese Iranian adolescents suffer from MUO. Thus, with a power of 80%, type I error of 0.05, desired CI of 0.95, and precision (*d*) of 7%, the minimum required sample size was calculated to be 188 subjects. A stratified, multistage cluster sampling design was used to randomly select the participants from 16 schools of five different districts of Isfahan, Iran. Then, only overweight and obese students [based on the growth curve of age-sex-specific BMI percentiles (28)] were invited to the current investigation. Participants with the following criteria were not included in this population-based study: (1) having genetic or endocrine disorders (e.g., type 1 diabetes mellitus, hypothyroidism, and Cushing's syndrome), (2) following a weight-loss diet, and (3) taking vitamin and mineral supplements and medications which might influence body weight, lipid profile, blood glucose, or hypertension. Finally, 203 overweight/obese adolescents (102 girls and 101 boys) aged 12 to <18 years were included in the current analysis. Written informed consent was obtained from each participant and his/her parents. The study protocol was ethically approved by the Local Ethics Committee of the Isfahan University of Medical Sciences.

### Assessment of Dietary Intakes

A validated 147-item food frequency questionnaire (FFQ) was used to assess the dietary intake of participants (29). Previous investigations showed that this FFQ could accurately indicate the relationship of dietary intakes with various diseases in Iranian adolescents (30, 31). Thus, this tool has reasonable validity and reliability for the assessment of foods and nutrients in the Iranian pediatric population. A trained nutritionist has completed FFQs and requested the individuals to report their frequency of consumption (based on daily, weekly, or monthly) and amount of consumption (based on standard common portion size) of food items. Then, the household measures (32) were used for conversion of the portion sizes of consumed foods in g/day. All food items were entered into Nutritionist IV software to compute the daily intake of energy and all nutrients.

### Assessment of DIL and DII

The FII is the area under the curve that has increased in insulin within 2 h after consumption of 1,000 kJ (239 kcal) of test food divided by the area under the curve after consumption of 1,000 kJ of reference food. The FII for each food item was obtained from published studies developed by Holt et al. (18), Bao et al. (33), and Bell et al. (34). For food items that their FIIs were not available in the food list of mentioned studies, FIIs of similar food items were used. The following formula was used for the calculation of the insulin load of each food:

Insulin load of a given food = FII of that food × energy content per 1 gram of that food (kcal) × amount of that food consumed (g/d) (35). For each participant, DIL was provided by summing the insulin load of all consumed foods. Then, the DII for each participant was computed by dividing the DIL by the total energy consumption of that person.

### Assessment of Anthropometric Indices and CMRFs

A trained nutritionist has measured all anthropometric values. A digital scale (Seca Instruments, Germany) was used to measure weight to the nearest 100 g when the subjects were wearing thin coats and no shoes. Height was measured with a stadiometer (to the nearest 0.1 cm), when the subject was standing with shoulders relaxed, without shoes. BMI was calculated based on the Quetelet formula [weight (kg)/height^2^ (m)], and subjects were classified as normal weight (5th < BMI <85th percentile), overweight (85th < BMI <95th percentile), and obese (BMI > 95th percentile) based on WHO growth curve of age-sex-specific BMI percentiles for adolescents (28). An unstretchable flexible anthropometric tape was used to measure waist circumference (WC) (accurate to 0.1 cm). The midway between the lowest rib and the superior border of the iliac crest was measured, after a normal expiration and without any pressure on the body surface. Measurement was repeated (2 times for each student), and an average of them was considered as WC value. A mercury sphygmomanometer with a suitable cuff size was used for the measurement of systolic BP (SBP) and diastolic BP (DBP). All BP measurements were conducted twice for each subject, at the right arm, after a rest period of 15 min. After 12 h overnight fast, blood samples were collected from all participants in a sitting position, according to the standard protocol, stored in vacuum tubes, and centrifuged within 30–45 min after collection. Fasting blood glucose (FBG) concentration was measured on the day of blood collection with an enzymatic colorimetric method by using glucose oxidase (Pars Azmoon commercial kits, Tehran, Iran). Serum insulin was measured using ELISA kits (Diagnostic Biochem Canada Inc.). In addition, Homeostasis Model Assessment Insulin Resistance (HOMA-IR) was calculated to estimate IR by using the following formula: HOMA-IR = [(fasting insulin (mU/L) × FBG (mmol/L)]/22.5. Serum high-density lipoprotein cholesterol (HDL-c) concentrations were measured with phosphotungstic acid, after precipitation of the apolipoprotein B-containing lipoproteins (Pars Azmoon commercial kits, Tehran, Iran). Serum triglyceride concentrations were also assayed using triacylglycerol kits by enzymatic colorimetric tests with glycerol phosphate oxidase (Pars Azmoon commercial kits, Tehran, Iran).

### Assessment of Metabolic Status

In the current analysis, two classification strategies were applied to determine the metabolic risk (MR) status: MHO vs. MUO. In the first strategy (MR_IDF_), the MUO was defined according to the International Diabetes Federation (IDF) criteria (36). Such that, obese individuals with at least two following criteria were considered as MUO, and the other obese subjects without the following criteria were considered as MHO: increased TG (≥150 mg/dl), decreased HDL-c (<40 mg/dl for the age of <16 years, and <50 mg/dl in women/ <40 mg/dl in men for the age of ≥16 years), increased FBG (≥100 mg/dl), and increased BP (≥130/85 mmHg). The second strategy (MR_IDF/HOMA−IR_) was a combination of the first strategy and the presence of IR based on HOMA-IR (37). In this strategy, obese individuals with at least two mentioned metabolic disorders and HOMA-IR > 3.16 were considered as MUO, while those with none or one of the mentioned cardiometabolic risk factors and HOMA-IR <3.16 were considered as MHO. The cutoff value of 3.16 was selected based on some earlier studies on obese children and adolescents who were in puberty age (38–40).

### Assessment of Other Variables

The validated Physical Activity Questionnaire for Adolescents (PAQ-A) was used for evaluating the level of physical activity in students (41). This questionnaire contains nine items, in which the first eight items are about the usual activity of adolescents and the ninth item is about unusual activity in the last week. After the calculation of total scores, individuals were classified as sedentary (or not having an orderly week activity) (score <2), low active (2 ≤ score <3), and active (score ≥ 3). A demographic questionnaire was applied to gather the information about age, gender, history of diseases, use of medications, and dietary supplements of students. The socioeconomic status (SES) was evaluated through a validated questionnaire which assessed the following variables: parental job, family size, parental education level, having cars in the family, having computers/laptops, having personal room, and taking trips in the year (42).

### Statistical Analysis

The normality of quantitative variables was examined using the Kolmogorov-Smirnov test. Continuous variables were presented as mean ± SD/SE and qualitative variables as frequency (percentage). Individuals were categorized based on tertiles of DIL and DII. The chi-square test and one-way ANOVA were, respectively, used to determine categorical and continuous variables across tertiles of DIL and DII. Furthermore, energy, age, and sex-adjusted dietary intakes of subjects across tertiles of DIL and DII were achieved using analysis of covariance (ANCOVA). ORs for MUO (based on IDF and IDF/HOMA-IR definitions) across tertiles of DIL and DII were examined using binary logistic regression in crude and multivariable-adjusted models. In the first model, adjustments were made for energy, sex, and age. In the second model, physical activity and SES were additionally adjusted. In the third model, BMI was added to the adjustments. In all models, the first tertile of DIL or DII was considered as the reference category. Tertiles of DIL and DII were treated as ordinal variables in logistic regression models for determining the trends. Stratified analysis based on the BMI category of participants was additionally carried out. All analyses were performed by using SPSS version 20 software. The value of *p* < 0.05 was considered statistically significant.

## Results

In the current analysis, data of 203 overweight or obese adolescents (50.2% girls) with a mean age of 13.98 (±1.61) years and an average BMI of 28.54 (±3.91) kg/m^2^ were examined. Among them, 38.9% (*n* = 79; 37 boys and 42 girls) of individuals based on the first strategy (MR_IDF_) and 33.0% (*n* = 67; 35 boys and 32 girls) of individuals based on the second strategy (MR_IDF/HOMA−IR_) suffered from MUO. In contrast, 61.1% (*n* = 124; 64 boys and 60 girls) and 67.0% (*n* = 136; 66 boys and 70 girls) of subjects were, respectively, classified as MHO, according to the first (MR_IDF_) and second strategies (MR_IDF/HOMA−IR_).

General characteristics and cardiometabolic risk factors (CMRFs) of study participants in the categories of DIL and DII are shown in Table 1. When we distributed participants across tertile of DIL, there were significant differences in all demographic variables and cardiometabolic variables across tertile of DIL, except for distribution of age (*p* = 0.32), SES (*p* = 0.13), and diastolic BP (*p* = 0.05); such that, individuals in the third tertile of DIL had a higher level of weight, BMI, WC, and CMRFs, and lower physical activity. In addition, those in the top tertile of DII had lower physical activity (*p* < 0.01) and HDL-c (*p* = 0.007), and higher FBG (*p* < 0.01), insulin (*p* = 0.003), HOMA-IR (*p* = 0.001), and TG (*p* < 0.001).

**Table 1:** General characteristics and cardiometabolic factors of study participants across tertiles of DIL and DII^a^.

	**Tertiles of DIL**				**Tertiles of DII**			
	**T1 (*n* = 67)**	**T2 (*n* = 68)**	**T3 (*n* = 68)**	***P*-value^b^**	**T1 (*n* = 67)**	**T2 (*n* = 68)**	**T3 (*n* = 68)**	***P*-value^b^**
Range	<103493.05	103827.72–124480.66	>124729.04	-	<39.16	39.19–41.47	>41.58	-
Sex, *n* (%)				<0.001				0.32
Boys	14 (20.9)	33 (48.5)	54 (79.4)		38 (56.7)	33 (48.5)	30 (44.1)	
Girls	53 (79.1)	35 (51.5)	14 (20.6)		29 (43.3)	35 (51.5)	38 (55.9)	
Age (year)	14.21 ± 0.20	13.91 ± 0.20	13.81 ± 0.17	0.32	14.01 ± 0.20	13.99 ± 0.19	13.93 ± 0.18	0.94
Weight (kg)	67.88 ± 0.96	71.596 ± 1.13	80.87 ± 1.56	<0.001	71.19 ± 1.43	75.29 ± 1.42	73.91 ± 1.34	0.11
Height (cm)	160.35 ± 0.79	162.33 ± 0.80	168.16 ± 1.02	<0.001	163.19 ± 0.94	164.43 ± 0.98	163.26 ± 0.97	0.59
BMI (kg/m^2^)	26.38 ± 0.30	27.12 ± 0.33	28.54 ± 0.47	<0.001	26.62 ± 0.39	27.77 ± 0.40	27.65 ± 0.37	0.07
Waist circumference (cm)	87.19 ± 0.74	88.65 ± 0.77	95.09 ± 1.06	<0.001	88.87 ± 1.12	90.93 ± 0.89	91.16 ± 0.84	0.18
Physical activity levels, *n* (%)				0.02				<0.001
Sedentary	25 (37.3)	29 (42.6)	35 (51.5)		12 (17.9)	35 (51.5)	42 (61.8)	
Low active	22 (32.8)	27 (39.7)	28 (41.2)		32 (47.8)	25 (36.8)	20 (29.4)	
Active	20 (29.9)	12 (17.6)	5 (7.4)		23 (34.3)	8 (11.8)	6 (8.8)	
Socioeconomic status^c^, *n* (%)				0.13				0.79
Low	17 (25.4)	23 (33.8)	19 (27.9)		17 (25.4)	23 (33.8)	19 (27.9)	
Medium	34 (50.7)	32 (47.1)	24 (35.3)		33 (49.3)	27 (39.7)	30 (44.1)	
High	16 (23.9)	13 (19.1)	25 (36.8)		17 (25.4)	18 (26.5)	19 (27.9)	
Systolic blood pressure (mmHg)	106.61 ± 2.84	112.72 ± 1.39	118.69 ± 2.01	0.001	111.97 ± 1.31	110.63 ± 2.89	115.50 ± 2.16	0.28
Diastolic blood pressure (mmHg)	70.83 ± 1.59	74.26 ± 0.81	75.34 ± 1.56	0.05	73.46 ± 0.83	71.98 ± 1.85	75.03 ± 1.24	0.29
Fasting blood glucose level (mg/dl)	95.48 ± 0.85	97.68 ± 1.22	101.21 ± 0.86	<0.001	93.85 ± 0.76	99.51 ± 1.07	100.97 ± 1.04	<0.001
Insulin (μUI/ml)	14.37 ± 0.65	20.10 ± 1.34	26.70 ± 1.93	<0.001	16.72 ± 1.08	20.43 ± 1.43	24.05 ± 1.86	0.003
HOMA-IR index	3.41 ± 0.16	4.91 ± 0.36	6.70 ± 0.48	<0.001	3.91 ± 0.26	5.09 ± 0.39	6.02 ± 0.46	0.001
Triglycerides (mg/dl)	94.36 ± 5.68	124.69 ± 8.07	146.40 ± 8.91	<0.001	102.61 ± 6.38	112.81 ± 7.94	150.15 ± 8.67	<0.001
HDL-c (mg/dl)	46.52 ± 0.89	45.06 ± 0.98	42.91 ± 0.96	0.02	46.99 ± 0.99	44.81 ± 0.93	42.71 ± 0.91	0.007

a *Values are mean ± SE; unless indicated. BMI, Body Mass Index; HOMA, Homeostasis Model Assessment Insulin Resistance; HDL-c, high-density lipoprotein cholesterol*.

b *P-value obtained from one way ANOVA and χ^2^ test for quantitative and categorical variables, respectively*.

c *Socioeconomic status (SES) score was evaluated based on parental education level, parental job, family size, having car in the family, having computer/laptop, having personal room, and taking trips by using a validated questionnaire*.

**Figure 1:**
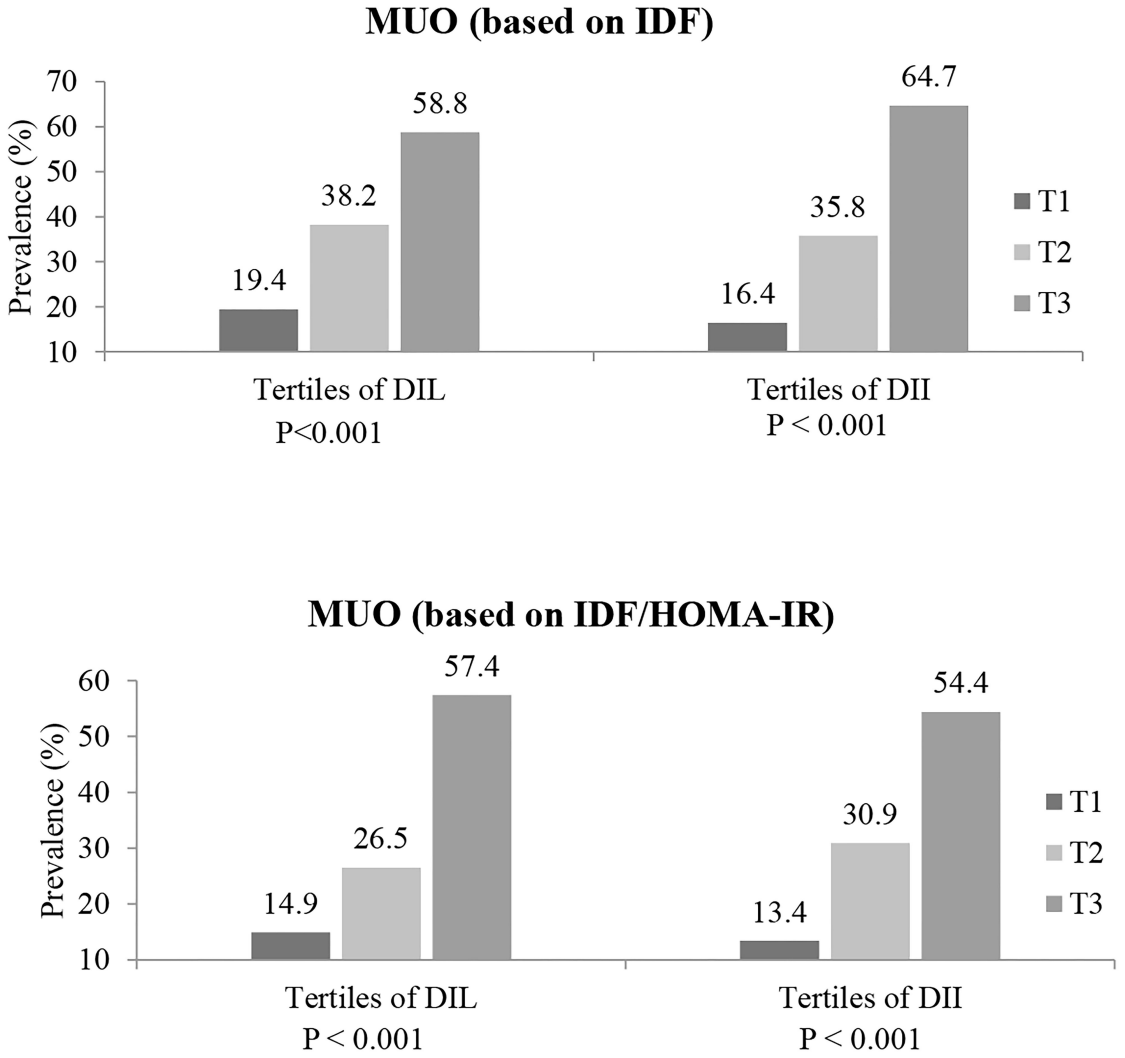
Prevalence of MUO (based on both IDF and IDF/HOMA-IR definitions) in tertiles of DIL and DII.

The prevalence of MUO (based on both definitions of IDF and IDF/HOMA-IR) in tertiles of DIL and DII is presented in Figure 1. Across tertile of DIL, 19.4, 38.2, and 58.8% of subjects in tertiles 1–3 were defined as MUO, based on IDF definition (*p* < 0.001). Similarly, based on IDF/HOMA-IR definition, 14.9, 26.5, and 57.4% of subjects across tertile of DIL were categorized as MUO (*p* < 0.001). Moreover, the prevalence of MUO was higher in the third tertile of DII, compared to the first category [based on IDF criteria (64.7 vs. 16.4%; *p* < 0.001); based on IDF/HOMA-IR definition (54.4 vs. 13.7%; *p* < 0.001)].

The dietary intakes of study participants based on tertiles of DIL and DII are indicated in Table 2. Individuals in the top tertile of DIL in comparison with the bottom tertile had a significantly lower intake of protein, fat, cholesterol, saturated fatty acid (SFA), monounsaturated fatty acids (MUFAs), polyunsaturated fatty acid (PUFA), vitamin C, vitamin A, riboflavin, vitamin B6, folate, vitamin B12, magnesium, zinc, total dietary fiber, and potassium. However, the intakes of energy, carbohydrate, thiamin, and niacin had an increasing trend across tertile of DIL, and there was no significant difference in the intakes of vitamin E, selenium, or sodium. Moreover, the intakes of protein, fat, cholesterol, MUFA, PUFA, vitamin C, vitamin A, riboflavin, vitamin B6, folate, vitamin B12, magnesium, zinc, total dietary fiber, and potassium were in a decreasing trend across tertile of DII. However, the intakes of carbohydrate, thiamin, selenium, and sodium had an increasing trend across tertile of DII, and there was no significant difference in the intakes of energy, SFA, niacin, and vitamin E.

**Table 2:** Dietary intakes (energy and macro/micro nutrients) of study participants across tertiles of DIL and DII^a^.

	**Tertiles of DIL**				**Tertiles of DII**			
	**T1 (*n* = 67)**	**T2 (*n* = 68)**	**T3 (*n* = 68)**	***P*-value^b^**	**T1 (*n* = 67)**	**T2 (*n* = 68)**	**T3 (*n* = 68)**	***P*-value^b^**
Range	<103493.05	103827.72–124480.66	>124729.04	-	<39.16	39.19–41.47	>41.58	-
Mean	93393.21	112822.37	142330.74	-	36.97	40.38	43.50	-
Energy, kcal	2513.27 ± 60.23	2818.60 ± 55.85	3311.75 ± 60.20	<0.001	2772.89 ± 65.98	2982.31 ± 65.20	2892.24 ± 65.41	0.08
Protein, % of energy	15.12 ± 0.25	14.12 ± 0.23	13.69 ± 0.25	0.01	14.83 ± 0.23	14.61 ± 0.23	13.48 ± 0.23	<0.001
Carbohydrate, % of energy	55.20 ± 0.62	58.71 ± 0.58	60.91 ± 0.62	<0.001	55.39 ± 0.57	58.54 ± 0.57	60.90 ± 0.57	<0.001
Fat, % of energy	31.34 ± 0.64	28.47 ± 0.60	26.75 ± 0.64	<0.001	31.43 ± 0.59	28.30 ± 0.59	26.84 ± 0.59	<0.001
Cholesterol, mg	321.69 ± 13.52	290.52 ± 11.53	234.56 ± 13.88	<0.001	313.08 ± 11.86	270.29 ± 11.71	263.28 ± 11.68	0.007
SFA, gr	29.76 ± 0.80	27.46 ± 0.68	24.86 ± 0.82	0.001	28.54 ± 0.71	26.90 ± 0.70	26.62 ± 0.70	0.12
MUFA, gr	31.68 ± 0.91	27.15 ± 0.78	23.86 ± 0.94	<0.001	30.85 ± 0.79	26.80 ± 0.78	25.03 ± 0.78	<0.001
PUFA, gr	30.79 ± 1.12	28.76 ± 0.95	25.93 ± 1.15	0.03	31.74 ± 0.93	28.20 ± 0.92	25.55 ± 0.92	<0.001
Vitamin C, mg	162.91 ± 8.05	131.90 ± 6.87	106.50 ± 8.27	<0.001	150.17 ± 7.13	130.74 ± 7.04	120.21 ± 7.02	0.01
Vitamin A, RAE	1325.29 ± 88.68	1043.15 ± 75.62	957.03 ± 91.05	0.02	1248.95 ± 77.12	1091.84 ± 76.13	983.56 ± 75.93	0.05
Thiamin, mg	2.47 ± 0.04	2.69 ± 0.03	2.76 ± 0.04	<0.001	2.48 ± 0.03	2.66 ± 0.03	2.78 ± 0.036	<0.001
Riboflavin, mg	2.63 ± 0.07	2.28 ± 0.06	1.99 ± 0.07	<0.001	2.46 ± 0.06	2.30 ± 0.06	2.14 ± 0.06	0.004
Niacin, mg	26.01 ± 0.48	28.05 ± 0.41	28.61 ± 0.49	0.001	26.76 ± 0.42	27.87 ± 0.41	28.05 ± 0.41	0.06
Vitamin B6, mg	1.86 ± 0.05	1.62 ± 0.04	1.38 ± 0.05	<0.001	1.72 ± 0.05	1.59 ± 0.05	1.54 ± 0.05	0.04
Vitamin E, mg	30.95 ± 1.64	30.76 ± 1.40	29.36 ± 1.69	0.79	32.42 ± 1.41	30.21 ± 1.39	28.46 ± 1.39	0.14
Folate, mcg	393.28 ± 12.93	306.23 ± 11.03	251.48 ± 13.28	<0.001	360.30 ± 11.72	314.76 ± 11.57	275.44 ± 11.54	<0.001
Vitamin B12, mcg	5.05 ± 0.20	4.54 ± 0.17	3.71 ± 0.21	<0.001	4.86 ± 0.17	4.46 ± 0.17	3.97 ± 0.17	0.002
Magnesium, mg	331.68 ± 8.20	286.86 ± 6.99	246.64 ± 8.42	<0.001	308.59 ± 7.54	287.16 ± 7.45	269.09 ± 7.43	0.001
Zinc, mg	12.13 ± 0.29	10.72 ± 0.25	9.06 ± 0.30	<0.001	11.25 ± 0.27	10.69 ± 0.27	9.97 ± 0.26	0.005
Selenium, mcg	0.08 ± 0.004	0.09 ± 0.003	0.09 ± 0.004	0.09	0.08 ± 0.003	0.09 ± 0.003	0.10 ± 0.003	<0.001
Total fiber, gr	22.31 ± 0.64	19.53 ± 0.55	16.53 ± 0.66	<0.001	21.39 ± 0.57	19.34 ± 0.56	17.62 ± 0.56	<0.001
Sodium, mg	4076.92 ± 163.91	3839.49 ± 139.76	4051.14 ± 168.28	0.42	3718.70 ± 139.53	3957.14 ± 137.73	4286.44 ± 137.37	0.01
Potassium, mg	4004.73 ± 117.62	3316.81 ± 100.29	2821.11 ± 120.76	<0.001	3689.71 ± 107.16	3371.30 ± 105.78	3077.02 ± 105.50	<0.001

a *Values are mean ± SE. Energy intake and macronutrients were adjusted for age and gender; all other values were adjusted for age, gender, and energy intake*.

b *P-value obtained from ANCOVA test for adjustment of energy intake*.

*SFA, Saturated fatty acids; MUFA, Monounsaturated fatty acids; PUFA, Polyunsaturated fatty acids*.

Multivariate adjusted OR and 95% CI for MUO across tertiles of DIL and DII are presented in Table 3. Adolescents in the top tertile of DIL had higher OR of MUO, compared to those in the bottom tertile, based on both IDF (OR = 5.93, 95% CI: 2.73–12.87) and IDF/HOMA-IR definitions (OR = 7.66, 95% CI: 3.35–17.51) in the crude model. After adjustments for potential confounders, this relationship remained significant. As a result, OR values of MUO in the highest tertile of DIL were 8.44 (for IDF definition: OR: 8.44; 95% CI: 2.24–31.78) and 5.86 (for IDF/HOMA-IR definition: OR: 5.86; 95% CI: 1.39–24.58) times more than the reference category, after considering all potential confounders. Furthermore, higher DII was significantly related to elevated OR of MUO based on IDF (OR = 9.33, 95% CI: 4.12–21.09) and IDF/HOMA-IR definitions (OR = 7.69, 95% CI: 3.29–17.97) in the crude model. After taking potential confounders into account, adolescents in the top tertile of DII, compared to the bottom tertile, had higher OR of MUO based on both definitions (for IDF definition: OR: 6.93; 95% CI: 2.59–18.57; for IDF/HOMA-IR definition: OR: 5.26; 95% CI: 1.85–14.97). In addition, OR of MUO across tertiles of DIL and DII had significant decreasing trends. We also reanalyzed the data and made adjustment for potential confounders one by one, as reported in Supplementary Table 1. The significance status of results was not changed while adjusting one by one for potential confounders.

**Table 3:** Multivariate adjusted odds ratio (OR) and 95% CI for MUO across tertiles of DIL and DII^a^.

	**Tertiles of DIL**				**Tertiles of DII**			
	**T1 (*n* = 67)**	**T2 (*n* = 68)**	**T3 (*n* = 68)**	***P*-trend**	**T1 (*n* = 67)**	**T2 (*n* = 68)**	**T3 (*n* = 68)**	***P*-trend**
**MUO phenotype based on IDF criteria**								
Cases (*n*)	13	26	40		11	24	44	
Crude	1.00	2.57 (1.18, 5.60)	5.93 (2.73, 12.87)	<0.001	1.00	2.77 (1.22, 6.27)	9.33 (4.12, 21.09)	<0.001
Model 1^b^	1.00	3.67 (1.55, 8.69)	13.12 (4.22, 40.71)	<0.001	1.00	2.44 (1.03, 5.75)	9.84 (4.16, 23.30)	<0.001
Model 2^c^	1.00	3.04 (1.16, 7.97)	9.00 (2.48, 32.65)	0.001	1.00	1.51 (0.58, 3.94)	6.90 (2.60, 18.35)	<0.001
Model 3^d^	1.00	2.95 (1.11, 7.81)	8.44 (2.24, 31.78)	0.002	1.00	1.47 (0.56, 3.89)	6.93 (2.59, 18.57)	<0.001
**MUO phenotype based on HOMA-IR criteria**								
Cases (*n*)	10	18	39		9	21	37	
Crude	1.00	2.05 (0.86, 4.85)	7.66 (3.35, 17.51)	<0.001	1.00	2.87 (1.20, 6.87)	7.69 (3.29, 17.97)	<0.001
Model 1^b^	1.00	2.44 (0.96, 6.21)	11.58 (3.49, 38.44)	<0.001	1.00	2.44 (0.96, 6.14)	8.18 (3.32, 20.19)	<0.001
Model 2^c^	1.00	1.76 (0.62, 5.00)	7.11 (1.75, 28.89)	0.007	1.00	1.50 (0.53, 4.20)	5.19 (1.87, 14.41)	0.001
Model 3^d^	1.00	1.59 (0.55, 4.58)	5.86 (1.39, 24.58)	0.01	1.00	1.42 (0.49, 4.11)	5.26 (1.85, 14.97)	0.001

a *All values are odds ratios and 95% CI*.

b *Model 1: Adjusted for age, sex, and energy intake*.

c *Model 2: Additionally adjusted for physical activity and socioeconomic status (e.g., parental education, parental job, number of family members, having car in the family, having computer/laptop, having personal room, and taking trip)*.

d *Model 3: Additionally adjusted for body mass index (BMI)*.

The relationship of DIL and DII with OR of MUO, stratified by BMI status of participants, is presented in Table 4. In the crude model, obese subjects in the third tertile of DIL had higher OR of MUO based on both IDF (OR: 7.50; 95% CI: 2.14–26.24) and IDF/HOMA-IR definitions (OR: 6.82; 95% CI: 1.95–23.78), compared with the obese participants in the first tertile. After adjustment for potential confounder, this relationship strengthened; such that, obese subjects in the top tertile of DIL in comparison with the bottom tertile were, respectively, 15.03 (OR: 15.03; 95% CI: 2.48–90.84) and 12.47 (OR: 12.47; 95% CI: 2.07–74.81) times more likely to be MUO based on IDF and IDF/HOMA-IR definitions. Among overweight subjects, this association was significant in the crude model; but the relationship disappeared in fully adjusted model [based on IDF (OR: 4.90; 95% CI: 0.38–63.07) and IDF/HOMA-IR (OR: 1.28; 95% CI: 0.08–19.17)]. With regard to DII, obese participants in the third tertile in comparison with those in the first tertile had significantly higher OR of MUO according to IDF (OR: 12.88; 95% CI: 3.42–48.56) and IDF/HOMA-IR definitions (OR: 15.86; 95% CI: 3.82–65.90) in the crude model. This relationship strengthened after considering all confounders; such that, obese adolescents in the highest DII category, compared to the lowest level, were 22.52 (OR: 22.52; 95% CI: 4.21–120.40) and 35.79 (OR: 35.79; 95% CI: 5.53–231.48) times more likely to be MUO based on IDF and IDF/HOMA-IR definitions, respectively. In overweight participants, a significant association was found between DII and MUO based on IDF in the crude model, but no significant association was found in fully adjusted model (based on IDF: OR: 3.32; 95% CI: 0.73–14.96). In overweight subjects, although there was no significant association between DII and MUO based on IDF/HOMA-IR criteria in both crude and fully adjusted models, we found a nonsignificant increasing OR in the crude model) OR: 2.92; 95% CI: 0.91–9.33), while after considering all confounders, a nonsignificant lowering OR was obtained (OR: 0.59; 95% CI: 0.11–3.24). The OR of MUO (based on both IDF and IDF/HOMA-IR definitions) across tertiles of DIL and DII had a significant decreasing trend, only in obese participants. Furthermore, we considered the effect of potential confounders one by one, as reported in Supplementary Table 2; the significant status of findings was not changed in obese participants. However, in overweight subjects, the significant relationships were disappeared after adjustment for energy intake or physical activity.

**Table 4:** Multivariate adjusted odds ratio (OR) and 95% CI for MUO across tertiles of energy-adjusted DIL and DII, stratified by BMI categories^a^.

	**Tertiles of DIL**				**Tertiles of DII**			
	**T1**	**T2**	**T3**	***P*-trend**	**T1**	**T2**	**T3**	***P*-trend**
**MUO phenotype based on IDF criteria**								
Overweight (cases/ participants)	9/47	9/35	10/22	-	7/47	6/27	15/30	-
Crude	1.00	1.46 (0.51, 4.17)	3.51 (1.16, 10.67)	0.03	1.00	1.63 (0.48, 5.48)	5.71(1.94, 16.75)	0.002
Model 1^b^	1.00	2.77 (0.74, 10.33)	17.44 (2.08, 145.72)	0.01	1.00	1.45 (0.40, 5.24)	6.09 (1.93, 19.24)	0.002
Model 2^c^	1.00	2.70 (0.51, 14.31)	4.90 (0.38, 63.07)	0.20	1.00	0.77 (0.15, 3.80)	3.32 (0.73, 14.96)	0.08
Obese (cases/participants)	4/20	17/33	30/46	-	4/20	18/41	29/38	-
Crude	1.00	4.25 (1.16, 15.45)	7.50 (2.14, 26.24)	0.002	1.00	3.13 (0.89, 11.01)	12.88 (3.42, 48.56)	<0.001
Model 1^b^	1.00	4.88 (1.25, 18.95)	11.64 (2.35, 57.56)	0.003	1.00	2.89 (0.77, 10.79)	16.83 (3.98, 71.16)	<0.001
Model 2^c^	1.00	5.29 (1.17, 23.79)	15.03 (2.48, 90.84)	0.003	1.00	2.68 (0.62, 11.54)	22.52 (4.21, 120.40)	<0.001
**MUO phenotype based on IDF /HOMA-IR criteria**								
Overweight (cases/participants)	6/47	4/35	10/22	-	6/47	5/27	9/30	-
Crude	1.00	0.88 (0.22, 3.39)	5.69 (1.71, 18.89)	0.007	1.00	1.55 (0.42, 5.67)	2.92 (0.91, 9.33)	0.07
Model 1^b^	1.00	1.00 (0.21, 4.77)	8.39 (0.92, 76.17)	0.07	1.00	1.61 (0.41, 6.31)	2.85 (0.84, 9.58)	0.09
Model 2^c^	1.00	0.55 (0.08, 3.82)	1.28 (0.08, 19.17)	0.91	1.00	0.58 (0.10, 3.44)	0.59 (0.11, 3.24)	0.57
Obese (cases/ participants)	4/20	14/33	29/46	-	3/20	16/41	28/38	-
Crude	1.00	2.94 (0.80, 10.76)	6.82 (1.95, 23.78)	0.002	1.00	3.62 (0.91, 14.39)	15.86 (3.82, 65.90)	<0.001
Model 1^b^	1.00	3.25 (0.83, 12.65)	9.99 (2.00, 49.93)	0.004	1.00	3.39 (0.78, 14.62)	23.09 (4.74, 112.35)	<0.001
Model 2^c^	1.00	3.15 (0.72, 13.79)	12.47 (2.07, 74.81)	0.005	1.00	3.46 (0.68, 17.52)	35.79 (5.53, 231.48)	<0.001

a *All values are odds ratios and 95% confidence intervals*.

b *Model 1: Adjusted for age, sex, and energy intake*.

c *Model 2: Additionally adjusted for physical activity and socioeconomic status (e.g., parental education, parental job, number of family members, having car in the family, having computer/laptop, having personal room, and taking trip)*.

The relationship of DIL and DII with OR of MUO, stratified by gender of participants, is presented in Table 5. Comparing the highest vs. lowest tertile of DIL, there was a significant relationship between DIL and OR of MUO in girls in the crude model (based on IDF: OR: 18.46; 95% CI: 3.64–93.51 and based on IDF/HOMA-IR: OR: 25.8; 95% CI: 4.96, 134.0). However, this relationship disappeared in maximally adjusted model (based on IDF: OR: 9.86; 95% CI: 0.66, 147.1 and based on IDF/HOMA-IR: OR: 3.80; 95% CI: 0.22, 63.4). As there was no boy with MUO in the first tertile of DIL, the software could not report OR for MUO in boys.

**Table 5:** Multivariate adjusted odds ratio (OR) and 95% CI for MUO across tertiles of energy-adjusted DIL and DII, stratified by sex^a^.

	**Tertiles of DIL**				**Tertiles of DII**			
	**T1**	**T2**	**T3**	***P*-trend**	**T1**	**T2**	**T3**	***P*-trend**
**MUO phenotype based on IDF criteria**								
Girl (Cases)	13	17	12	-	6	11	25	-
Crude	1.00	2.90 (1.16, 7.23)	18.46 (3.64, 93.51)	<0.001	1.00	1.75 (0.55, 5.53)	7.37 (2.40, 22.61)	<0.001
Model 1^b^	1.00	2.84 (0.84, 9.57)	18.11(1.72, 190.34)	0.01	1.00	2.09 (0.59, 7.40)	10.24 (2.91, 36.08)	<0.001
Model 2^c^	1.00	2.66 (0.63, 11.11)	8.03 (0.58, 110.9)	0.10	1.00	1.12 (0.25, 4.94)	6.07 (1.31, 28.17)	0.007
Model 3^d^	1.00	2.81 (0.66, 11.9)	9.86(0.66,147.1)	0.08	1.00	1.21(0.27, 5.43)	6.60 (1.38, 31.57)	0.006
Boy (Cases)	0^e^	9	28	-	5	13	19	
Crude	-	-	-	<0.001	1.00	4.29 (1.33, 13.84)	11.40 (3.43, 37.78)	<0.001
Model 1	-	-	-	0.002	1.00	3.39 (1.01,11.40)	10.92 (3.20,37.24)	<0.001
Model 2	-	-	-	0.01	1.00	2.39 (0.64, 8.94)	8.40 (2.21, 31.89)	0.002
Model 3	-	-	-	0.02	1.00	2.78 (0.65, 11.88)	13.46 (2.87, 63.17)	0.001
**MUO phenotype based on IDF /HOMA-IR criteria**								
Girl (Cases)	10	10	12	-	4	9	19	-
Crude	-	1.72 (0.62, 4.70)	25.8 (4.96, 134.0)	<0.001	1.00	2.16 (0.59, 7.93)	6.25 (1.82, 21.43)	0.002
Model 1	-	1.26 (0.33,4.84)	12.99 (1.14,146.95)	0.08	1.00	2.35 (0.55, 9.97)	8.07 (2.05, 31.78)	0.002
Model 2	-	0.74 (0.15, 3.66)	4.33 (0.27, 67.21)	0.45	1.00	1.08 (0.19,5.89)	3.20 (0.60, 17.11)	0.08
Model 3	-	0.71(0.14, 3.57)	3.80 (0.22, 63.4)	0.54	1.00	1.001(0.17, 5.62)	2.92 (0.52, 16.27)	0.10
Boy (Cases)	0^e^	8	27	-	5	12	18	-
Crude	-	-	-	<0.001	1.00	3.77 (1.16, 12.24)	9.90 (3.0, 32.57)	<0.001
Model 1	-	-	-	0.003	1.00	2.86 (0.83, 9.78)	9.61 (2.80, 32.94)	<0.001
Model 2	-	-	-	0.01	1.00	2.04 (0.53, 7.81)	7.45 (1.92, 28.92)	0.003
Model 3	-	-	-	0.02	1.00	2.36 (0.54, 10.25)	11.62 (2.45,55.00)	0.001

a *All values are odds ratios and 95% CI*.

b *Model 1: Adjusted for age, sex, and energy intake*.

c *Model 2: Additionally adjusted for physical activity and socioeconomic status (e.g., parental education, parental job, number of family members, having car in the family, having computer/laptop, having personal room, and taking trip)*.

d *Model 3: Additionally adjusted for body mass index (BMI)*.

e *As there was no boy with MUO in first tertile of DIL, software could not report OR of MUO in boys*.

The DII was significantly related to OR of MUO based on IDF criteria in both genders in crude model and adjustment model for all confounders. In addition, there was a significant relationship between DII and OR of MUO based on IDF/HOMA-IR criteria in boys in crude and fully adjusted models. Although DII was significantly related to OR of MUO based on IDF/HOMA-IR criteria in girls in the crude model (OR: 6.25; 95% CI: 1.82, 21.43), this association was removed, after adjustment for all potential confounders (OR: 2.92; 95% CI: 0.52, 16.27). As presented in Supplementary Table 3, we controlled the effect of potential confounders one by one and found that the significant association in girls disappeared after adjustment for physical activity.

## Discussion

We found that more adherence to a diet with high DIL or DII was associated with higher OR of MUO (based on both IDF and IDF/HOMA-IR criteria), with an increasing trend in Iranian adolescents, especially in obese ones. In overweight students, these associations were not independent of other covariates. To the best of our knowledge, this is the first investigation that evaluated the association of DIL and DII with metabolic health status in adolescents.

The current analysis showed that a high percentage of Iranian overweight/obese adolescents (33.0–38.9% based on different definitions) suffered from MUO. MHO, as an intermediate state before the development of MUO, maybe a temporary and not safe state; however, it is a worthy first step in the management of obesity and its cardiometabolic complications. To delay the onset of obesity-related metabolic comorbidities, adolescents could be clinically advised to improve their diet quality and aware of foods with high insulinemic potential to decrease their consumption.

Similar to our study, several previous studies have investigated the relationship between dietary patterns and risk of metabolic disease in children and adolescents. A cross-sectional study on 137 European overweight/obese adolescents has documented that adherence to the Mediterranean diet was related to a low risk of MUO (43). In addition, a 16-week intervention with a Mediterranean diet has protectively affected CMR, including lean mass, BMI, fat mass, glucose, HDL-c, TG, total cholesterol (TC), and low-density lipoprotein cholesterol (LDL-c) in obese children and adolescents (44). The SEARCH Nutrition Ancillary Study was also conducted on adolescents with type 1 diabetes and showed that Mediterranean diet was protectively linked to better glycemic control and lipid profile in both cross-sectional and longitudinal analyses (45). In addition, 1 year dietary intervention with a Mediterranean diet has significantly decreased the TC and LDL-c levels in hypercholesterolemic children (46). In contrast, prior evidence showed that Western dietary pattern was related to an increased risk of MetS and obesity in children and adolescents (47–49). A cross-sectional study that investigated 205 overweight and 146 normal-weight 6-year Turkish children has documented that higher DII and DIL, especially for breakfast and dinner, were related to higher OR of overweight (25). In the current analysis, we demonstrated that more adherence to DIL or DII was strongly related to increased OR of MUO, especially in obese adolescents.

Previous studies have suggested some pathways to explain the mechanisms underlying the relationship of DIL and DII with OR of metabolic disorders. Diet with high insulinemic potential could increase insulin secretion and, consequently, increase carbohydrate oxidation and decrease fat oxidation; therefore, such a diet could enhance fat storage, especially in the abdominal area, and increase the risk of abdominal obesity and metabolically unhealthy profile (50). Moreover, as potentially high insulinemic foods have a high rate of digestion, absorption, and transformation to glucose, these foods would rapidly increase the blood glucose and blood insulin and, consequently, decrease glucose excursion (51). The rapid decrease in blood glucose would result in reducing satiety, restoring hunger sensation, and, consequently, increasing the calorie intake, which could lead to an increased risk of abdominal obesity and metabolically unhealthy profile (51, 52). Furthermore, higher DIL and DII are related to IR (53) and low C-peptide concentration, respectively, which maybe the results of β-cell dysfunction (35). Unfortunately, in this study, we could not evaluate C-peptide concentration, due to a limited research budget.

In the current analysis, the relationship of DIL and DII with MUO was associated for the first time in adolescents, and using two various definitions of metabolic health status could strengthen the findings. Moreover, the effect of potential confounders was controlled in the analyses. However, there were some limitations that should be kept in mind while interpreting the findings. Since the study had a cross-sectional design, it was impossible to define causality. Further prospective studies are needed to confirm the causality. The study sample was selected from overweight and obese adolescents, so the findings could only be extrapolated to overweight and obese adolescents and could not be generalized to the whole children population. Although a validated FFQ was used for the assessment of dietary intake, recall bias and other potential reporting biases were inevitable and might affect the findings. Despite adjustments for several potential confounders, some confounders, such as degree of pubertal development, sleep deprivation, food habits, birth weight, and parental obesity, were not evaluated; therefore, the residual effects of such confounders or other unknown or unmeasured confounders might influence the findings. Finally, BMI and WC were measured, but body composition and fat distribution that would be important confounders and would involve in metabolic health status were not evaluated.

In conclusion, the current population-based cross-sectional study revealed that higher DIL and DII were strongly related to increased OR of MUO in Iranian adolescents, especially in obese participants. Further investigations, especially with prospective design, are needed to affirm these findings.

## Data Availability Statement

The raw data supporting the conclusions of this article will be made available by the authors, without undue reservation.

## Ethics Statement

The study protocol was approved by the Local Ethics Committee of Isfahan University of Medical Sciences. Written informed consent to participate in this study was provided by the participants' legal guardian/next of kin.

## Author Contributions

ZH, SM, AA, MA, and PS contributed to conception, design, data collection, data interpretation, manuscript drafting, and approval of the final version of the manuscript, and agreed for all aspects of the work. All authors contributed to the article and approved the submitted version.

## Funding

The financial support for conception, design, data analysis, and manuscript drafting comes from the Food Security Research Center, Isfahan University of Medical Sciences, Isfahan, Iran.

## Conflict of Interest

The authors declare that the research was conducted in the absence of any commercial or financial relationships that could be construed as a potential conflict of interest.

## Publisher's Note

All claims expressed in this article are solely those of the authors and do not necessarily represent those of their affiliated organizations, or those of the publisher, the editors and the reviewers. Any product that may be evaluated in this article, or claim that may be made by its manufacturer, is not guaranteed or endorsed by the publisher.

## Abbreviations

FFQ: food frequency questionnaire
OR: odds ratios
95% CI: 95% confidence interval
GBD: Global Burden of Disease
BMI: body mass index
MHO: metabolically healthy obese
MUO: metabolically unhealthy obese
CMRF: cardiometabolic risk factors
IDF: International Diabetes Federation
HOMA-IR: Homeostasis Model Assessment Insulin Resistance
IR: insulin resistance
PAQ-A: Physical Activity Questionnaire for Adolescents
SES: socioeconomic status
MUFA: monounsaturated fatty acids
PUFA: polyunsaturated fatty acids
SFA: saturated fatty acids
WHO: World Health Organization
WC: waist circumference
BP: blood pressure
SBP: systolic blood pressure
DBP: diastolic blood pressure
TG: triglycerides
HDL-c: high density lipoprotein cholesterol
FBG: fasting blood glucose
ANOVA: analysis of variance
ANCOVA: analysis of covariance
SPSS: statistical package for the social sciences
SD: standard deviation
SE: standard error
FII: Food Insulin Index
DIL: dietary insulin load
DII: dietary insulin index
MetS: metabolic syndrome
T2D: type 2 diabetes mellitus
CVD: cardiovascular disease
MR: metabolic risk.
