# Supplementary material for: Association of Dietary Insulin Index and Dietary Insulin Load With Metabolic Health Status in Iranian Overweight and Obese Adolescents
Source: Front Nutr. 2022 Mar 17;9:821089. doi: 10.3389/fnut.2022.821089 (PMC8969564; doi:10.3389/fnut.2022.821089)
Supplement: Supplementary file 1 [file Table_1.docx]

| **Supplemental Table 1. Multivariate adjusted odds ratio (OR) and 95% confidence interval (CI) for MUO across tertiles of DIL and DII ^a^.** | | | | | | | | | | | | | |  |
| --- | --- | --- | --- | --- | --- | --- | --- | --- | --- | --- | --- | --- | --- | --- |
|  | | Tertiles of DIL | | | | | | | Tertiles of DII | | | | | |
|  | T1  (n=67) | | T2  (n=68) | | T3  (n=68) | | P-trend | | T1  (n=67) | T2  (n=68) | T3  (n=68) | | P-trend |  |
| **MUO phenotype based on IDF criteria** | | | | | | |  | |  |  |  | |  |  |
| Cases (n) | 13 | | 26 | | 40 | |  | | 11 | 24 | 44 | |  |  |
| Crude | 1.00 | | 2.57 (1.18, 5.60) | | 5.93 (2.73, 12.87) | | <0.001 | | 1.00 | 2.77 (1.22, 6.27) | 9.33 (4.12, 21.09) | | <0.001 |  |
| Model 1^b^ | 1.00 | | 2.76 (1.25, 6.08) | | 6.59 (2.97, 14.58) | | <0.001 | | 1.00 | 2.82 (1.24, 6.43) | 9.75 (4.27, 22.26) | | <0.001 |  |
| Model 2^c^ | 1.00 | | 3.82 (1.65, 8.84) | | 14.59 (5.35, 39.81) | | <0.001 | | 1.00 | 2.87 (1.26, 6.55) | 10.04 (4.36, 23.10) | | <0.001 |  |
| Model 3^d^ | 1.00 | | 3.67 (1.55, 8.69) | | 13.12 (4.22, 40.71) | | <0.001 | | 1.00 | 2.44 (1.03, 5.75) | 9.84 (4.16, 23.30) | | <0.001 |  |
| Model 4^e^ | 1.00 | | 3.27 (1.26, 8.46) | | 8.90 (2.53, 31.27) | | 0.001 | | 1.00 | 1.68 (0.65, 4.31) | 6.08 (2.62, 17.62) | | <0.001 |  |
| Model 5^f^ | 1.00 | | 3.04 (1.16, 7.97) | | 9.00 (2.48, 32.65) | | 0.001 | | 1.00 | 1.51 (0.58, 3.94) | 6.90 (2.60, 18.35) | | <0.001 |  |
| Model 6^g^ | 1.00 | | 2.95 (1.11, 7.81) | | 8.44 (2.24, 31.78) | | 0.002 | | 1.00 | 1.47 (0.56, 3.89) | 6.93 (2.59, 18.57) | | <0.001 |  |
| **MUO phenotype based on HOMA-IR criteria** | | | | | | |  | |  |  |  | |  |  |
| Cases (n) | | 10 | | 18 | | 39 | |  | 9 | 21 | | 37 |  | |
| Crude | | 1.00 | | 2.05 (0.86, 4.85) | | 7.66 (3.35, 17.51) | | <0.001 | 1.00 | 2.87 (1.20, 6.87) | | 7.69 (3.29, 17.97) | <0.001 | |
| Model 1^b^ | | 1.00 | | 2.10 (0.88, 5.01) | | 7.98 (3.46, 18.42) | | 0.003 | 1.00 | 2.88 (1.20, 6.89) | | 7.73 (3.30, 18.09) | 0.001 | |
| Model 2^c^ | | 1.00 | | 2.70 (1.10, 6.64) | | 15.04 (5.37, 42.14) | | <0.001 | 1.00 | 3.02 (1.25, 7.26) | | 8.40 (3.53, 19.96) | <0.001 | |
| Model 3^d^ | | 1.00 | | 2.44 (0.96, 6.21) | | 11.58 (3.49, 38.44) | | <0.001 | 1.00 | 2.44 (0.96, 6.14) | | 8.18 (3.32, 20.19) | <0.001 | |
| Model 4^e^ | | 1.00 | | 1.99 (0.72, 5.47) | | 7.28 (1.95, 27.13) | | <0.001 | 1.00 | 1.45 (0.52, 4.03) | | 4.69 (1.73, 12,72) | <0.001 | |
| Model 5^f^ | | 1.00 | | 1.76 (0.62, 5.00) | | 7.11 (1.75, 28.89) | | 0.007 | 1.00 | 1.50 (0.53, 4.20) | | 5.19 (1.87, 14.41) | 0.001 | |
| Model 6^g^ | | 1.00 | | 1.59 (0.55, 4.58) | | 5.86 (1.39, 24.58) | | 0.01 | 1.00 | 1.42 (0.49, 4.11) | | 5.26 (1.85, 14.97) | 0.001 | |
| a All values are odds ratios and 95% confidence intervals.  b Model 1: Adjusted for age.  c Model 2: Additionally adjusted for sex.  d Model 3: Additionally adjusted for energy intake.  e Model 4: Additionally adjusted for and physical activity.  f Model 5: Additionally adjusted for socioeconomic status (parental education, parental job, number of family members, having car in the family, having computer/laptop, having personal room and having trip).  g Model 6: Additionally adjusted for body mass index (BMI). | | | | | | | | | | | | | |  |

| **Supplemental Table 2. Multivariate adjusted odds ratio (OR) and 95% confidence interval (CI) (by ) for MUO across tertiles of energy-adjusted DIL and DII, stratified by BMI ^a^.** | | | | | | | | | |
| --- | --- | --- | --- | --- | --- | --- | --- | --- | --- |
|  |  | Tertiles of DIL | | | | Tertiles of DII | | | |
|  |  | T1 | T2 | T3 | P-trend | T1 | T2 | T3 | P-trend |
| MUO phenotype based on IDF criteria | | | |  |  |  |  |  |  |
| **Overweight**  **(Cases)** | | 9 | 9 | 10 | - | 7 | 6 | 15 | - |
| Crude | | 1.00 | 1.46 (0.51, 4.17) | 3.51 (1.16, 10.67) | 0.03 | 1.00 | 1.63 (0.48, 5.48) | 5.71(1.94, 16.75) | 0.002 |
| Model 1^b^ | | 1.00 | 1.67 (0.56, 4.9) | 5.20 (1.55, 17.40) | 0.009 | 1.00 | 1.57 (0.45, 5.38) | 6.04 (1.99, 18.31) | 0.002 |
| Model 2^c^ | | 1.00 | 2.49 (0.77, 8.05) | 13.51 (2.68, 68.11) | 0.002 | 1.00 | 1.43 (0.40, 5.08) | 5.96 (1.96, 18.12) | 0.002 |
| Model 3^d^ | | 1.00 | 2.77 (0.74, 10.33) | 17.44 (2.08, 145.72) | 0.01 | 1.00 | 1.45 (0.40, 5.24) | 6.09 (1.93, 19.24) | 0.002 |
| Model 4^e^ | | 1.00 | 2.23 (0.49, 10.09) | 4.99 (0.44, 56.57) | 0.18 | 1.00 | 0.63 (0.13, 2.97) | 2.45 (0.60, 9.95) | 0.14 |
| Model 5^f^ | | 1.00 | 2.70 (0.51, 14.31) | 4.90 (0.38, 63.07) | 0.20 | 1.00 | 0.77 (0.15, 3.80) | 3.32 (0.73, 14.96) | 0.08 |
| **Obese**  **(Cases)** | | 4 | 17 | 30 | - | 4 | 18 | 29 | - |
| Crude | | 1.00 | 4.25 (1.16, 15.45) | 7.50 (2.14, 26.24) | 0.002 | 1.00 | 3.13 (0.89, 11.01) | 12.88 (3.42, 48.56) | <0.001 |
| Model 1^b^ | | 1.00 | 4.20 (1.15, 15,33) | 7.27 (2.07, 25.57) | 0.002 | 1.00 | 3.16 (0.89, 11.22) | 13.31 (3.48, 50.80) | <0.001 |
| Model 2^c^ | | 1.00 | 5.06 (1.32, 19.26) | 12.90 (2.97, 56.00) | 0.001 | 1.00 | 3.14 (0.88, 11.20) | 15.12 (3.81, 59.91) | <0.001 |
| Model 3^d^ | | 1.00 | 4.88 (1.25, 18.95) | 11.64 (2.35, 57.56) | 0.003 | 1.00 | 2.89 (0.77, 10.79) | 16.83 (3.98, 71.16) | <0.001 |
| Model 4^e^ | | 1.00 | 4.55 (1.09, 19.0) | 11.66 (2.14, 63.52) | 0.005 | 1.00 | 2.53 (0.59, 10.80) | 16.50 (3.36, 80.91) | <0.001 |
| Model 5^f^ | | 1.00 | 5.29 (1.17, 23.79) | 15.03 (2.48, 90.84) | 0.003 | 1.00 | 2.68 (0.62, 11.54) | 22.52 (4.21, 120.40) | <0.001 |
| MUO phenotype based on IDF /HOMA-IR criteria | | | | |  |  |  |  |  |
| **Overweight**  **(Cases)** | | 6 | 4 | 10 | - | 6 | 5 | 9 | - |
| Crude | | 1.00 | 0.88 (0.22, 3.39) | 5.69 (1.71, 18.89) | 0.007 | 1.00 | 1.55 (0.42, 5.67) | 2.92 (0.91, 9.33) | 0.07 |
| Model 1^b^ | | 1.00 | 0.95 (0.24, 3.71) | 7.44 (2.05, 26.94) | 0.004 | 1.00 | 1.15 (0.41, 5.56) | 2.91 (0.91, 9.32) | 0.07 |
| Model 2^c^ | | 1.00 | 1.14 (0.27, 4.71) | 11.43 (2.29, 57.03) | 0.003 | 1.00 | 1.72 (0.44, 6.65) | 3.02 (0.93, 9.77) | 0.06 |
| Model 3^d^ | | 1.00 | 1.00 (0.21, 4.77) | 8.39 (0.92, 76.17) | 0.07 | 1.00 | 1.61 (0.41, 6.31) | 2.85 (0.84, 9.58) | 0.09 |
| Model 4^e^ | | 1.00 | 0.56 (0.09, 3.34) | 1.55 (0.11, 20.91) | 0.85 | 1.00 | 0.52 (0.09, 3.05) | 0.57 (0.10, 3.01) | 0.55 |
| Model 5^f^ | | 1.00 | 0.55 (0.08, 3.82) | 1.28 (0.08, 19.17) | 0.91 | 1.00 | 0.58 (0.10, 3.44) | 0.59 (0.11, 3.24) | 0.57 |
| **Obese**  **(Cases)** | | 4 | 14 | 29 | - | 3 | 16 | 28 | - |
| Crude | | 1.00 | 2.94 (0.80, 10.76) | 6.82 (1.95, 23.78) | 0.002 | 1.00 | 3.62 (0.91, 14.39) | 15.86 (3.82, 65.90) | <0.001 |
| Model 1^b^ | | 1.00 | 2.91 (0.79,10.67) | 6.66 (1.90, 23.32) | 0.002 | 1.00 | 3.65 (0.91, 14.59) | 16.28 (3.88, 68.31) | <0.001 |
| Model 2^c^ | | 1.00 | 3.46 (0.91, 13.16) | 11.96 (2.76, 51.73) | 0.001 | 1.00 | 3.63 (0.90, 14.57) | 18.98 (4.34, 83.02) | <0.001 |
| Model 3^d^ | | 1.00 | 3.25 (0.83, 12.65) | 9.99 (2.00, 49.93) | 0.004 | 1.00 | 3.39 (0.78, 14.62) | 23.09 (4.74, 112.35) | <0.001 |
| Model 4^e^ | | 1.00 | 2.91 (0.69, 12.16) | 9.98 (1.83, 54.45) | 0.007 | 1.00 | 3.41 (0.66, 17.41) | 26.89 (4.46, 162.15) | <0.001 |
| Model 5^f^ | | 1.00 | 3.15 (0.72, 13.79) | 12.47 (2.07, 74.81) | 0.005 | 1.00 | 3.46 (0.68, 17.52) | 35.79 (5.53, 231.48) | <0.001 |
| a All values are odds ratios and 95% confidence intervals.  b Model 1: Adjusted for age.  c Model 2: Additionally adjusted for sex.  d Model 3: Additionally adjusted for energy intake.  e Model 4: Additionally adjusted for and physical activity.  f Model 5: Additionally adjusted for socioeconomic status (parental education, parental job, number of family members, having car in the family, having computer/laptop, having personal room and having trip). | | | | | | | | | |

| **Supplemental Table 3. Multivariate adjusted odds ratio (OR) and 95% confidence interval (CI) for MUO across tertiles of energy-adjusted DIL and DII, stratified by sex ^a^.** | | | | | | | | | | | | | | | | |
| --- | --- | --- | --- | --- | --- | --- | --- | --- | --- | --- | --- | --- | --- | --- | --- | --- |
|  | Tertiles of DIL | | | | | | | | Tertiles of DII | | | | | | | |
|  | T1 | | T2 | | T3 | | P-trend | | T1 | | T2 | T3 | | | P-trend | |
| MUO phenotype based on IDF criteria | | | | |  | |  | |  | |  |  | | |  | |
| **Girl**  **(Cases)** | 13 | | 17 | | 12 | | - | | 6 | | 11 | 25 | | | - | |
| Crude | 1.00 | | 2.90 (1.16, 7.23) | | 18.46 (3.64, 93.51) | | <0.001 | | 1.00 | | 1.75 (0.55, 5.53) | 7.37 (2.40, 22.61) | | | <0.001 | |
| Model 1^b^ | 1.00 | | 2.85 (1.14, 7.13) | | 18.24 (3.59, 92.51) | | <0.001 | | 1.00 | | 1.89 (0.59, 6.05) | 7.99 (2.54, 25.08) | | | <0.001 | |
| Model 2^c^ | 1.00 | | 2.84 (0.84, 9.57) | | 18.11(1.72, 190.34) | | 0.01 | | 1.00 | | 2.09 (0.59, 7.40) | 10.24 (2.91, 36.08) | | | <0.001 | |
| Model 3^d^ | 1.00 | | 2.40 (0.59, 9.68) | | 7.94 (0.59, 106.5) | | 0.11 | | 1.00 | | 1.03 (0.24, 4.46) | 5.15 (1.18, 22.44) | | | 0.009 | |
| Model 4^e^ | 1.00 | | 2.66 (0.63, 11.11) | | 8.03 (0.58, 110.9) | | 0.10 | | 1.00 | | 1.12 (0.25, 4.94) | 6.07 (1.31, 28.17) | | | 0.007 | |
| Model 5^f^ | 1.00 | | 2.81 (0.66, 11.9) | | 9.86(0.66,147.1) | | 0.08 | | 1.00 | | 1.21(0.27, 5.43) | 6.60 (1.38, 31.57) | | | 0.006 | |
| **Boy**  **(Cases)** | 0 ^g^ | | 9 | | 28 | | - | | 5 | | 13 | 19 | | |  | |
| Crude | - | | - | | - | | <0.001 | | 1.00 | | 4.29 (1.33, 13.84) | 11.40 (3.43, 37.78) | | | <0.001 | |
| Model 1^b^ | - | | - | | - | | 0.001 | | 1.00 | | 4.25 (1.31, 13.84) | 12.22 (3.61, 41.35) | | | <0.001 | |
| Model 2^c^ | - | | - | | - | | 0.002 | | 1.00 | | 3.39 (1.01,11.40) | 10.92 (3.20,37.24) | | | <0.001 | |
| Model 3^d^ | - | | - | | - | | 0.01 | | 1.00 | | 2.35 (0.64, 8.61) | 8.02 (2.18, 29.52) | | | 0.001 | |
| Model 4^e^ | - | | - | | - | | 0.01 | | 1.00 | | 2.39 (0.64, 8.94) | 8.40 (2.21, 31.89) | | | 0.002 | |
| Model 5^f^ | - | | - | | - | | 0.02 | | 1.00 | | 2.78 (0.65, 11.88) | 13.46 (2.87, 63.17) | | | 0.001 | |
| MUO phenotype based on IDF /HOMA-IR criteria | | | | | | |  | |  | |  |  | | |  | |
| **Girl**  **(Cases)** | | 10 | | 10 | | 12 | | - | | 4 | 9 | | 19 | - | |  |
| Crude | | 1.00 | | 1.72 (0.62, 4.70) | | 25.8 (4.96, 134.0) | | <0.001 | | 1.00 | 2.16 (0.59, 7.93) | | 6.25 (1.82, 21.43) | 0.002 | |  |
| Model 1^b^ | | 1.00 | | 1.79 (0.64, 4.93) | | 26.87 (5.12, 140.9) | | <0.001 | | 1.00 | 2.14 (0.58, 7.92) | | 6.21 (1.80, 21.4) | 0.002 | |  |
| Model 2^c^ | | 1.00 | | 1.26 (0.33,4.84) | | 12.99 (1.14,146.95) | | 0.08 | | 1.00 | 2.35 (0.55, 9.97) | | 8.07 (2.05, 31.78) | 0.002 | |  |
| Model 3^d^ | | 1.00 | | 0.71 (0.15, 3.36) | | 3.98 (0.26, 59.6) | | 0.50 | | 1.00 | 1.06 (0.19, 5.82) | | 3.21 (0.60, 17.06) | 0.07 | |  |
| Model 4^e^ | | 1.00 | | 0.74 (0.15, 3.66) | | 4.33 (0.27, 67.21) | | 0.45 | | 1.00 | 1.08 (0.19,5.89) | | 3.20 (0.60, 17.11) | 0.08 | |  |
| Model 5^f^ | | 1.00 | | 0.71(0.14, 3.57) | | 3.80 (0.22, 63.4) | | 0.54 | | 1.00 | 1.001(0.17, 5.62) | | 2.92 (0.52, 16.27) | 0.10 | |  |
| **Boy**  **(Cases)** | | 0 ^g^ | | 8 | | 27 | | - | | 5 | 12 | | 18 | - | |  |
| Crude | | - | | - | | - | | <0.001 | | 1.00 | 3.77 (1.16, 12.24) | | 9.90 (3.0, 32.57) | <0.001 | |  |
| Model 1^b^ | | - | | - | | - | | 0.001 | | 1.00 | 3.74 (1.14, 12.32) | | 10.89 (3.21, 36.87) | <0.001 | |  |
| Model 2^c^ | | - | | - | | - | | 0.003 | | 1.00 | 2.86 (0.83, 9.78) | | 9.61 (2.80, 32.94) | <0.001 | |  |
| Model 3^d^ | | - | | - | | - | | 0.01 | | 1.00 | 1.98 (0.53, 7.39) | | 6.94 (1.87, 25.67) | 0.003 | |  |
| Model 4^e^ | | - | | - | | - | | 0.01 | | 1.00 | 2.04 (0.53, 7.81) | | 7.45 (1.92, 28.92) | 0.003 | |  |
| Model 5^f^ | | - | | - | | - | | 0.02 | | 1.00 | 2.36 (0.54, 10.25) | | 11.62 (2.45,55.00) | 0.001 | |  |
| a All values are odds ratios and 95% confidence intervals.  b Model 1: Adjusted for age.  c Model 2: Additionally adjusted for energy intake.  d Model 3: Additionally adjusted for and physical activity.  e Model 4: Additionally adjusted for socioeconomic status (parental education, parental job, number of family members, having car in the family, having computer/laptop, having personal room and having trip).  f Model 5: Additionally adjusted for body mass index (BMI).  g As there were no boys with MUO in first tertile of DIL, software could not report OR of MUO in boys. | | | | | | | | | | | | | | | | |
